# Molecular genetics and genomics generate new insights into invertebrate pest invasions

**DOI:** 10.1111/eva.12071

**Published:** 2013-04-24

**Authors:** Heather Kirk, Silvia Dorn, Dominique Mazzi

**Affiliations:** ^1^ ETH Zurich Institute of Agricultural Sciences, Applied Entomology Zurich Switzerland; ^2^ Present address: University of Zurich Institute of Systematic Botany Zurich Switzerland

**Keywords:** adaptation, agroecosystems, dispersal, insects, pest management, range expansion

## Abstract

Invertebrate pest invasions and outbreaks are associated with high social, economic, and ecological costs, and their significance will intensify with an increasing pressure on agricultural productivity as a result of human population growth and climate change. New molecular genetic and genomic techniques are available and accessible, but have been grossly underutilized in studies of invertebrate pest invasions, despite that they are useful tools for applied pest management and for understanding fundamental features of pest invasions including pest population demographics and adaptation of pests to novel and/or changing environments. Here, we review current applications of molecular genetics and genomics in the study of invertebrate pest invasions and outbreaks, and we highlight shortcomings from the current body of research. We then discuss recent conceptual and methodological advances in the areas of molecular genetics/genomics and data analysis, and we highlight how these advances will further our understanding of the demographic, ecological, and evolutionary features of invertebrate pest invasions. We are now well equipped to use molecular data to understand invertebrate dispersal and adaptation, and this knowledge has valuable applications in agriculture at a time when these are critically required.

## Introduction

Invertebrate *pests* (see Box 1 for definitions of terms shown in italics) are ubiquitous, damaging, and often insidious components of anthropogenic and natural landscapes and are responsible for immense economic losses worldwide; for example, Pimentel et al. (2005) estimated that in the United States (USA) alone, invertebrate crop pests are associated with more than $14 billion in annual costs. Governments, nonprofit organizations, and industries continue to make enormous investments to develop appropriate measures to prevent, mitigate, and reduce the impact of pests. It is timely to review the contribution of molecular genetics and genomics to understanding pest invasions for a number of reasons. First, global human population increases are expected to dramatically intensify pressure on agricultural systems over the next 40 years, and controlling pest infestations is integral to meeting ongoing challenges in food security (Godfray et al. 2010; Thrall et al. 2011). Second, climate change is predicted to have large effects on agricultural productivity and natural ecosystems, in part as a result of changing dynamics between plants and their pests, which may include geographic range expansions by pest species, and increasing density of pest populations (Gregory et al. 2009; Gornall et al. 2010; Thrall et al. 2011). Third, advances in molecular genetics and genomics are yielding new and affordable tools for understanding demographic and adaptive processes in a variety of species (Barrett and Hoekstra 2011; Ekblom and Galindo 2011; Kirk and Freeland 2011), and these tools have been underutilized in the study of invertebrate pest species.

Box 1 **Definitions**

**Adaptation:** An evolutionary process that occurs as a result of natural selection. Adaptation allows an organism to become better suited to living under a particular set of environmental conditions. Alternatively used to refer to a trait that has evolved by means of natural selection.

**Adaptive (or non‐neutral) molecular markers:** Markers that are associated with genes or regulatory regions involved in adaptation. Adaptive, non‐neutral markers provide information about evolutionary processes that result from selection.

**DNA barcode:** A short species‐specific sequence of DNA that can be used for diagnostics purposes to identify an unknown sample to the species level.

**Invasive species:** Species that have been introduced from their native range to one or more non‐native areas and cause significant economic or ecological damage. In some cases, populations within species vary in their propensity for invasions (i.e., some introduced populations may cause significant ecological or economic damage, while others do not).

**Neutral molecular markers:** Markers that are not associated with genes or regulatory regions involved in adaptation. Neutral markers provide information about evolutionary processes other than selection, such as migration and genetic drift.

**Pest species:** Species that disrupt an ecosystem, causing significant ecological or economic damage.

**Phenotypic plasticity:** The ability of an individual to express different phenotypes in response to variation in environmental conditions.

**Preadaptation:** A pre‐existing structure or trait that predisposes a population or species to adapt in response to a novel selection pressure.

Invertebrate species, including exotic invasive and native pests, are increasingly recognized as a major management concern. A recent review (Kenis et al. 2009) showed that of 403 primary research publications that investigated the ecological impacts of invasive alien insects, 60% were published between 2000 and 2007, indicating that this is a relatively new and expanding area of research. However, these studies cumulatively incorporated only 72 insect species, and 32% of the studies concerned two ant species (*Solenopsis invicta* and *Linepithema humile*), which emphasizes the need to expand the taxonomic coverage of invertebrate pests.

There are a number of applied and fundamental challenges regarding invertebrate pest invasions and outbreaks in anthropogenic and natural ecosystems. Applied challenges include the early detection of pest invasions and outbreaks, the development and assessment of control measures, and the improvement in predictive models that can allow policy makers and practitioners to evaluate the risk associated with pest species. The need for appropriate strategies to meet these challenges is highlighted by several pest invasions that have occurred during the last decade. The spotted wing Drosophila (*Drosophila suzukii*) was collected for the first time in the USA in 2008 (invasion history reviewed by Hauser 2011), and correct identification to the species level was not made for more than a year; the species continues to be frequently confused with the western cherry fruit fly (*Rhagoletis indifferens*). Initial observations of this *Drosophila* species on berry crops did not raise major concern, because *Drosophila* species do not generally cause significant damage to crops in the USA (Hauser 2011). Although the species is assumed to have originated in South‐East Asia, its center of origin has not yet been identified, which may hamper attempts to identify suitable potential biological control agents from its native range. Similar problems have been reported from other recent invaders, including the marmorated stink bug (*Halyomorpha halys*) in the USA, which was first collected in the USA in 1996, but was not correctly identified until 2001 (Nielsen and Hamilton 2009 and references therein).

A fundamental understanding of the factors that contribute to invertebrate pest invasions and outbreaks is also lacking. Invasions and outbreaks by invertebrate pests share many characteristics with invasions by other species, and biological invasions have recently garnered considerable attention (Prentis et al. 2008; van Kleunen et al. 2010). *Invasive species* have been increasingly recognized as a major threat to regional and global biodiversity (Wagner and Van Driesche 2010) and are also useful model species for studies of rapid evolution, niche shifts, and range expansion (Prentis et al. 2008; Verhoeven et al. 2011). For these reasons, considerable effort has been invested to identify factors that may predispose species or populations to become successful invaders of non‐native or novel habitats (see Box 2).

Box 2 **Can existing inferences regarding invasive plant species be extrapolated to invertebrate pests?**

It is not clear whether existing hypotheses derived mostly based on data from invasive plants (e.g., Bossdorf et al. 2005; Mitchell et al. 2006; Schierenbeck and Ellstrand 2009; van Kleunen et al. 2010, 2011; Moles et al. 2012) are also broadly applicable to invertebrate pests (Hayes and Barry 2008). Are there factors that predispose invertebrate pests to invasions, and how are these different from factors that contribute to plant invasions?

While this is certainly not an exhaustive list, three factors are frequently suggested to contribute to invasions, at least in plants: First, high genetic diversity is associated with the potential for rapid adaptation to novel selection pressures in non‐native habitats (Schierenbeck and Ellstrand 2009), although it should be noted that neutral molecular variation is not necessarily a proxy for quantitative variation in life‐history traits or for adaptive potential (Reed and Frankham 2001). Second, high *phenotypic plasticity* or *preadaptation* to high‐resource uptake across environments confers high fitness in new or changing environments (e.g., van Kleunen et al. 2011; but see Palacio‐López and Gianoli 2011), and third, high ecological similarity between habitats in the native and introduced range of a species promotes the successful establishment of introduced species (i.e., habitat matching; Hayes and Barry 2008).

However, these factors may vary in importance with regard to invasions by invertebrate pests. Rapid adaptation and/or phenotypic plasticity may be less critical for invaders of agricultural systems compared with invaders of more natural landscapes, because the ‘new’ habitats that they encounter are typically more homogeneous in space and time, both in terms of the genotypic diversity of the host plants, and with regard to the abiotic conditions in which the host plants are grown. With regard to phenotypic plasticity, various invertebrate species exhibit a wide range of developmental and behavioral processes that are influenced by environmental cues, including sex determination, transitions between developmental stages, dispersal behaviors, and host preferences (reviewed by Simpson et al. 2011). In some cases, plastic responses may even limit the distribution range of invertebrate species. For example, Sobek‐Swant et al. (2012) showed that a loss of cold tolerance in response to acclimation to a mid‐winter warm period reduces the ability of the emerald ash borer (*Agrilus planipennis*) to tolerate subsequent cold periods. The range of this invasive species may therefore be limited by the occurrence of local climatic extremes during the winter season. The relative role of plastic responses compared with adaptive genomic change in pest invasions has barely been touched upon. Although much effort has been dedicated to understanding which traits make plants good invaders (reviewed by Moles et al. 2012), similar lines of research for invertebrate pests are lacking.

However, a large proportion of such studies, and the inferences that are derived from them, are based on data from invasive plants (reviewed by Bossdorf et al. 2005; Mitchell et al. 2006; Schierenbeck and Ellstrand 2009; Moles et al. 2012), and the majority of studies on invertebrates target species that use wild plants, rather than cultivated plants, as hosts (Kenis et al. 2009). While this review is not restricted to pests of agroecosystems, it is pertinent to note that pest invasions and outbreaks in agroecosystems can be qualitatively different from invasions of natural ecosystems; agricultural pests generally benefit from high levels of ecological homogeneity among and within different regions (Benton et al. 2003) relative to species that invade natural habitats, and often use host plants that possess little genetic variability compared with wild host species. Also, agricultural pests are sometimes subject to selection pressures that are unique to agroecosystems, such as pest control programs, seasonal crop harvests, and the effects of artificial (anthropogenic) selection on plant–insect interactions (see Harvey and Gols 2011; Tamiru et al. 2011 for examples of the latter).

We review applied and fundamental applications of molecular genetics/genomics in understanding invertebrate invasions and outbreaks. Wherever possible, we provide examples of invertebrate pests of cash crops because they are associated with a disproportionately high actual and potential economic impact; yet, little attention has been paid to the factors that facilitate invertebrate pest invasions and outbreaks compared with, for example, weed invasions (Kenis et al. 2009; Box 2). We exclude examples of vectors of human disease, as they have been reviewed extensively elsewhere (e.g., Veracx and Raoult 2012; Vontas et al. 2012; Williams 2012), and they involve factors (such as demographic history and genetic variation in resistance) that are beyond the scope of this review.

## Current applications of molecular genetics and genomics in studies of pest invasions

Molecular genetics approaches have been widely applied to the study of invertebrate pest species from a variety of taxa for at least 20 years and are indispensible tools for addressing applied and fundamental questions relating to invertebrate pest invasions. Primarily applied uses include the following: (i) the identification of pest species, (ii) the identification of mechanisms of pesticide resistance, and (iii) the assessment of the efficacy of pest management practices. Other molecular genetic applications address more fundamental questions regarding the biology of pest species or ultimate questions regarding pest species evolution. Predominantly putatively *neutral molecular markers* have been used, often in combination with historical records, (iv) to reconstruct invasion histories and colonization routes, and to understand demographic processes such as population bottlenecks and regional dispersal patterns, that are associated with pest invasions and outbreaks (see Table 1), or (v) to develop ecological and evolutionary hypotheses about mechanisms of pest invasions.

**Table 1:** Examples of recent studies that used molecular genetics to infer invasion histories of invertebrate pests

Pest species	Common name	Order	Host plant species	Molecular marker	Conclusions	References
*Linepithema humile*	Argentine ant	Hymenoptera	N/A	mtDNA (COI‐COII, cytB)	10 introduced haplotypes globally including five in Japan, indicative of multiple introductions to Japan. The dominant Japanese supercolony is identical to the dominant supercolony distributed throughout Europe, North America, and Australia. Two of the remaining Japanese haplotypes were likely introduced from the USA, and two were unique to Japan (putative source populations could not be inferred.)	Inoue et al. (2013)
*Grapholita molesta*	Oriental fruit moth	Lepidoptera	Stone and pome fruits, including peach, nectarines, cherries, apple, and pear	SSR	Little evidence for multiple introductions on each continent. Data suggest introductions from Asia to Australia and from North America to South Africa, South America, and the Azores. A recent, secondary introduction likely occurred from Brazil to Europe	Kirk et al. (2013)
*Megastigmus schimitscheki*	None (seed chalcid wasp species)	Hymenoptera	The ornamental cedar tree species *Cedrus atlantica* and *Cedrus brevifolia*	SSR, mtDNA (COI)	Cyprus was unambiguously the source of introduced *M. schimitscheki* in France. Invasion in non‐native area was associated with a severe bottleneck	Auger‐Rozenberg et al. (2012)
*Sirex noctilio*	None (woodwasp species)	Hymenoptera	Pine trees (*Pinus* sp.)	SSR, mtDNA (COI)	Two potential global invasion sources of *S. noctilio* were suggested. A nonsampled source population was likely introduced to South Africa and Chile, followed by a serial introduction from Chile to Switzerland. A second complex scenario involves three independent introductions from Europe to Oceania, South Africa, and South America followed by admixture among continents	Boissin et al. (2012)
*Tecia solanivora*	Potato tuber moth	Lepidoptera	Potato	mtDNA (cytB)	Evidence for stepwise introductions from north to south in Central and South America	Torres‐Leguizamón et al. (2011)
*Myzus persicae nicotianae*	Tobacco aphid	Homoptera	Tobacco	SSR	Evidence of multiple introductions from Europe to North America, and introductions from North America to South America. Loss of genetic diversity is associated with ongoing invasion. Also evidence for a highly successful ‘super clone’ in the Americas	Zepeda‐Paulo et al. (2010)
*Bactrocera cucurbitae*	Melon fly	Diptera	Polyphagous: melons, squash, tomato, bean, orange, etc.	SSR	Data suggest central Asian origin, continent‐scale differentiation, with low levels of long‐distance and complex inter‐regional dispersal	Virgilio et al. (2010)
*Ceratitis capitata*	Mediterranean fruit fly (medfly)	Diptera	Highly polyphagous	SSR, RAPDs, mtDNA	Evidence for invasion from Kenya to Mediterranean Basin (MB) and from MB to Latin America and the Pacific. Invasion was accompanied by a loss of genetic diversity	Reviewed by Malacrida et al. (2007)
*Scirtothrips perseae*	Avocado thrips	Thysanoptera	Avocado	mtDNA and SSRs	Recent introduction to California likely derived from a single‐source population from Coatepec Harinas, Mexico	Rugman‐Jones et al. (2007)

SSR, simple sequence repeat; AFLP, amplified fragment length polymorphism; mtDNA, mitochondrial DNA; cytB, cytochrome b gene; COI/COII, cytochrome c oxidase subunits I and II.

See main text for a detailed description of the invasion history of the western corn rootworm *Diabrotica virgifera* and the Asian ladybird *Harmonia axyridis* (not mentioned here to minimize redundancy).

### Identification of pest species

The accurate identification of pest species is a prerequisite for the deployment of appropriate management strategies; it is critical to identify pest species accurately at points of entry to a new geographic area or during the early phases of an invasion. Correct identification to the species level is also essential for the implementation of selective pest control measures. However, rapid identification of pests is often impaired by uninformative morphological traits and a lack of available molecular data, such as species‐specific *DNA barcodes*. Control of *D. suzukii*, a recent example of a recent cross‐continental invader (mentioned above), was hampered by the time lag between its initial sighting and its correct identification. During this time lag, the species spread from California to Oregon, Washington State, Florida, and British Columbia (Canada). Utilizing molecular tools when suspicions about the identity of this pest were first raised may have permitted early identification and implementation of quarantine and other pest control measures, preventing its rapid early spread.

Many other pest species are similarly difficult to identify based on morphology, especially because the frequently elusive immature stages are often responsible for the bulk of crop damage. Examples include economically damaging thrips species, which exhibit few diagnostic morphological characters, and are often mistaken for less destructive congeners. *Thrips palmi* likely originated in South‐East Asia, but is frequently intercepted on imports of bitter gourd, eggplant, and ornamental flowers from Asia to the Caribbean, West Africa, the USA, South America, Africa, Australia, and the United Kingdom (Cardona et al. 2002; Glover et al. 2010). DNA barcoding based on the cytochrome oxidase I (COI) gene permits this species to be rapidly distinguished from congeners and other morphologically similar species (Glover et al. 2010). Similarly, a number of tortricid moth species are internal pome and stone fruit feeders that occur sympatrically, and the larvae of these species are frequently misidentified based on morphology or feeding damage symptoms. Molecular markers based on polymorphisms in the COI gene can be used to distinguish between four pest species of the genera *Cydia* and *Grapholita* that have overlapping distributions in Europe (Chen and Dorn 2009). Such molecular markers provide useful tools that allow authorities at national entry ports to ensure that imported goods are free of pests, and also permit land managers to accurately identify pests from agricultural landscapes and to implement appropriate control measures.

### Identifying mechanisms of resistance to xenobiotics

Another application of molecular genetics in invertebrate invasion biology is the identification of mechanisms involved in resistance to xenobiotics, including pesticides and toxins expressed by transgenic crops. Resistance mechanisms have been well studied because of their direct and measurable economic consequences, and a number of gene families are known to be involved in the evolution of pesticide resistance across a number of taxa. They include genes that control the production of enzymes that break down xenobiotics such as P450 monooxygenases, esterases, and glutathione *S*‐transferases, and genes that reduce the binding of insecticides at target sites such as acetylcholinesterase, sodium channels, and GABA receptors (Hemingway and Ranson 2000). Identifying the genes underlying resistance allows to estimate the frequency of resistant genotypes across populations (Franck et al. 2007) and to develop pest control measures that circumvent prevalent resistance mechanisms (Gao et al. 2012). For example, the high combined frequencies of two alleles of the *para* gene that confer pyrethroid resistance to the diamondback moth (*Plutella xylostella*) over a wide area in southern Australia suggest that alternative pest control measures should be used for this species in the region (Endersby et al. 2011).

Moreover, recent comparative genomics work has suggested that some invertebrate species may be better preadapted than others to rapidly evolve pesticide resistance, based on their genomic architecture. The highly polyphagous and multivoltine two‐spotted spider mite (*Tetranychus urticae*) frequently evolves pesticide resistance in the field, and Grbić et al. (2011) suggested that high diversity in gene families involved in detoxification predisposes this species to rapid *adaptation* (discussed in greater detail in the Comparative genomics section below). Another major crop pest, the Colorado potato beetle (*Leptinotarsa decemlineata*), is native to North America and invasive in Europe and Asia, and has developed resistance to an astounding 52 different compounds from all major synthetic pesticide classes (reviewed by Alyokhin et al. 2008). A wide variety of different resistance mechanisms is involved, often within small geographic areas. This implies that there is high functional diversity of genes involved in resistance to xenobiotics in *L. decemlineata*, which is not surprising given that the species is specialized on a host family that produces a range of toxic alkaloids. Further genomics work on *L. decemlineata* and other pest species that demonstrate rapid and repeated evolution of pesticide resistance may allow us to better understand how and under what conditions pests adapt to pest management practices, including pesticide applications. If only one or a limited number of genome regions are involved in adaptation to insecticides in a particular pest species, then management of resistance genotypes may be relatively straightforward. However, data are accumulating from a number of pest species in which many genes and paralogous gene copies are involved in insecticide resistance, and some resistance alleles may provide cross‐resistance to multiple pesticides. If this is generally the case, then management of insecticide resistance can become extremely complex (e.g., Gao et al. 2012), particularly when several pests need to be managed simultaneously.

### Assessing the efficacy of management practices that target invasive pests

Population genetic data can be used to predict and measure the effects of different management practices (Mazzi and Dorn 2012), although to date, they have infrequently been used for this purpose. Measurements of immigration and gene flow between different management areas, and estimates of effective sizes of populations under different management regimes can be derived from molecular genetic data. Such an approach was used by Franklin et al. (2010) to assess the impacts of greenhouse management practices on cabbage looper (*Trichoplusia ni*) populations in British Columbia. The authors showed that yearly pesticide applications in greenhouses led to strong bottleneck effects and likely resulted in selection for pesticide‐resistant genotypes. A small number of resistant genotypes likely migrated between neighboring greenhouses in the spring. These results underscore the need for coordinated pest management efforts between growers to prevent the regional spread of resistant genotypes.

### Understanding pest demography and reconstructing invasion routes

Understanding pest dispersal and demography is beneficial because it permits the identification of new invasions, facilitates the monitoring of current infestations and the prevention of further ones, and aids in the planning and implementation of control measures (Hulme 2009; Estoup and Guillemaud 2010; Ridley et al. 2011). With regard to the latter point, identification of the center of origin of a species can, for example, yield natural enemies of the species that can be used as agents of biological control in integrated pest management programs. This may be an important step for the management of *D. suzukii* (discussed in *i* above), for which the native range has not yet been identified (Hauser 2011).

Most often putatively neutral microsatellite or amplified fragment length polymorphism markers are used, sometimes in combination with mitochondrial sequences, to reconstruct migration pathways, to quantify gene flow across different spatial scales, to identify migrants between different regions, and to estimate admixture between populations from different origins (Handley et al. 2011). Such an approach was recently used to reconstruct invasion routes of the oriental fruit moth (*Grapholita molesta*), which is a globally invasive pest of stone and pome fruit trees including peach and apple (Kirk et al. 2013). Eastern Asia was inferred to be the center of origin of this species, and molecular genetic data implied that the species has been introduced to Europe at least twice, including once from South America (Kirk et al. 2013).

Perhaps the most comprehensive example of invasion route reconstruction is that of the red fire ant (*S. invicta*), which was achieved using both nuclear and mitochondrial molecular markers (Ascunce et al. 2011). *Solenopsis invicta* builds large colonies that damage crop roots, and ants sometimes damage or kill young citrus trees by feeding on the bark, cambium, and shoots (Banks et al. 1991). More problematic is the damage that ants cause to property through mound‐building activities and the painful or even dangerous stings that they inflict on humans, pets, and livestock. The species originated in South America and is currently distributed in the USA, the Caribbean, Australia, New Zealand, and parts of Asia. Ascunce et al. (2011) sampled and genotyped ant colonies from different geographic locations throughout the current global range of the species. The authors found that at least nine separate introductions have occurred from the southern USA to other parts of the invaded range, and that recent introductions have not occurred from the native range in South America. This indicates that measures to prevent the global spread of the species should target USA ports of trade.

Studies of pest population dynamics *within* countries or regions can also be useful for developing management strategies. For example, the lepidopteran species *Cydia pomonella* is a major worldwide pest of fruit and nut species, such as apple, apricot, and walnut, but its dispersal abilities appear to vary according to landscape characteristics and management practices (Chen and Dorn 2010; Franck and Timm 2010). Chen and Dorn (2010) characterized 15 populations of *C. pomonella* in Switzerland. The authors showed that even proximately located (<10 km) populations were genetically differentiated, suggesting limited natural gene flow over small spatial scales in this region, even when significant barriers to dispersal were absent. The same study also identified a single population from an apple orchard close to the international airport in Zurich, which clustered separately from all other sampled populations in Switzerland; this finding implied a possible introduction from a foreign population via the airways, which in turn suggests that airport screening for fruits and/or pests should be more stringent.

By reconstructing the demographic history of pest outbreaks and introduction routes associated with invasions, we can more effectively minimize the movement of pests across landscapes and between geographic regions. Also, understanding the demographic characteristics and geographic pathways of pest dispersal and invasions is central to the development of ecological and evolutionary hypotheses regarding the mechanisms of pest invasions and outbreaks.

### Developing ecological and evolutionary hypotheses about the mechanisms of pest invasions

Although a number of hypotheses have been developed based on data from invasive plants regarding factors underlying invasions (see Box 2, Facon et al. 2006), it is debatable whether knowledge derived from plants can be generalized to invertebrate invasions. For example, a recent review (Pyšek et al. 2010) investigated the relationship between invasive species richness and biogeographic, climatic, economic, and human demographic factors across the European landscape. For invasive plants, 91% of the variation in species richness was explained by the minimal adequate model, which indicates that many primary factors that correlate with the number of invasive plant species in a landscape have already been identified, and these include human population density, wealth, and climate. In contrast, only 27% of the variation was explained for invasive insects, all of which was attributed to human factors such as population density and wealth. This suggests that there is still a large gap in our understanding of the factors that promote invertebrate invasions, both from a life‐history perspective (i.e., which life‐history characteristics are common to invasive invertebrate species?) and from a landscape perspective (i.e., which geographic, climatic, and human demographic features of a landscape make it more prone to species invasions?). Yet, filling this gap will be important for developing predictive models for forecasting and pest management purposes.

One example of a factor that is frequently claimed to be associated with invasions across many different taxa is the occurrence of multiple introductions across a particular geographic area (Dlugosch and Parker 2008; Prentis et al. 2008; Schierenbeck and Ellstrand 2009; Guillemaud et al. 2011). Multiple introductions of the western corn rootworm (*Diabrotica virgifera virgifera*), one of the most damaging and costly pests of maize in the USA (Miller et al. 2005; Ciosi et al. 2008), are linked to its invasion of Europe. *Diabrotica virgifera* originated in Central America and was first reported from the USA in 1867 and from Europe in 1992 (reviewed by Gray et al. 2009). Its successful establishment in Europe is associated with at least five introductions from the northern USA to Europe (Ciosi et al. 2008) although no secondary contact was inferred between populations derived from different origins (Ciosi et al. 2008). It is widely assumed that multiple introductions of pest species, followed by admixture between introduced populations, can reduce the potential for founder effects and may even increase the adaptive potential of introduced populations by generating recombinant genotypes that are better able to cope with novel environments (Dlugosch and Parker 2008; Prentis et al. 2008; Schierenbeck and Ellstrand 2009; Verhoeven et al. 2011). For example, Lombaert et al. (2010) showed that invasive European populations of the harlequin ladybird *Harmonia axyridis* were comprised of admixed populations from eastern North America and the local biocontrol strain derived from Asia. Subsequently, it was shown that hybrids between eastern North American and a biocontrol strain are fitter than either parental lineage with regard to a number of phenotypic traits including body size and generation time (Lombaert et al. 2011; Turgeon et al. 2011), which suggests that admixture may have partially fueled the invasion of Europe by the harlequin ladybird (Turgeon et al. 2011). Yet, evidence to support the hypothesis that admixture generally promotes invasiveness in invertebrates is scant. In contrast to plants, genetic diversity in insects is generally low in exotic invasive populations compared with native populations (Uller and Leimu 2011), which suggests that admixture may be more important for the former than for the latter. This again emphasizes the need to collect data from a wider selection of invertebrate taxa, which may further illuminate differences between the features of invasions by plants versus invertebrates.

Many invasions and outbreaks are thought to involve evolutionary change, including niche shifts and/or local adaptation (Prentis et al. 2008; Verhoeven et al. 2011). The most widely studied and best‐understood examples of local adaptation in invertebrate pest species include adaptation to exogenous and endogenous insecticides (including genetically modified crops; discussed above), and the evolution of genetically distinct host‐associated populations within species.

Work carried out on adaptation to insecticides has convincingly demonstrated that genetic structure at loci related to insecticide resistance can be shaped by pest control regimes and that structure at adaptive loci often differs compared with structure at putatively neutral loci. For example, the codling moth (*C. pomonella*) has developed resistance to insecticides in several parts of its range (Franck et al. 2007; and references therein). Franck et al. (2007) compared genetic structure of neutral loci with that of two loci that confer insecticide resistance across codling moth populations from 27 orchards. The frequencies of alleles that conferred resistance were positively correlated with the number of times each orchard was sprayed with insecticide annually, while only low levels of genetic structure were observed at the neutral loci. Insecticide applications likely represent a strong selection pressure that favors adaptive alleles at resistance loci, despite high migration rates between orchards, while high migration rates tend to homogenize genetic variation at loci that are not subject to strong selection (i.e., neutral loci).

Evidence of adaptation to different hosts was observed among populations of the cotton‐melon aphid (*Aphis gossypii*), a highly polyphagous pest. Carletto et al. (2009) identified five host races that were associated with Cucurbitaceae, cotton, eggplant, potato, and chili‐ or sweet pepper, and showed using supplementary plant transfer experiments that host races were somewhat specialized on their associated host plants. Local adaptation to different hosts in the field has also been demonstrated for the codling moth in Switzerland (Chen and Dorn 2010). Populations derived from apple, apricot, and walnut were genetically distinct from one another in a fruit‐growing valley in southwestern Switzerland. These studies demonstrate that pest populations can be structured according to the distribution of suitable host species across the landscape.

However, despite that some inroads have been made toward understanding the role of adaptive change in a limited number of pest invasions (see Table 2 for additional examples), there remains a paucity of data regarding the role of adaptation versus dispersal ability and other life‐history characteristics in the establishment and spread of invertebrate pests.

**Table 2:** Selected examples of recent studies that used molecular genetics to test for adaptation in invertebrate pests

Pest species	Common name	Order	Host plant species	Molecular marker	Conclusions	References
*Acyrthosiphon pisum*	Pea aphid	Hemiptera	More than 20 legume genera	SSR	Based on analysis of sympatric populations specialized on red clover, alfalfa, and pea. Eleven host race–associated outlier loci were identified out of 390 genotyped SSR loci, and these outlier loci were associated with several candidate genes, which may be involved in host adaptation, including genes encoding salivary proteins and chemosensory genes. Results support the hypothesis that adaptation to each host species occurred only once	Jaquiéry et al. (2012)
*Cydia pomonella*	Codling moth	Lepidoptera	Pome and stone fruits, and nuts	SSR	Evidence for significant population structure within Switzerland. Evidence for differentiation according to host plant species (apple, apricot, and walnut)	Chen and Dorn (2010)
*Trichoplusia ni*	Cabbage looper	Lepidoptera	Crucifers and other vegetables	AFLPs	Between *Bt* sprayed fields and greenhouse populations, no difference in genetic diversity, but lower differentiation in field compared with greenhouse. Significant IBD in both cases	Franklin et al. (2010)
*Schizaphis graminum*	Greenbug	Hemiptera	Wheat, barley, noncultivated grasses	SSR	Evidence for adaptation to host plant species, but also evidence of geographic differentiation	Weng et al. (2010)
*Frankliniella occidentalis*	Western flower thrips	Thysanoptera	Highly polyphagous	mtDNA and SSRs	2 ecotypes associated with climatic variation. One ecotype adapted to hot/dry habitat and the other ecotype adapted to cool/moist habitat	Brunner and Frey (2010)
*Aphis gossypii*	Cotton‐melon aphid	Hemiptera	Polyphagous, e.g., cucumber, cotton, eggplant, and okra	SSR	47% of variation in data explained by host plant, only 16% explained by geographic region: region was important within a host race, but host race was far more important overall	Carletto et al. (2009)
*Rhopalosiphum padi*	Bird cherry‐oat aphid	Hemiptera	Oat, brome wheat, barley, rye, other grasses	SSR	No significant effect of host plant. Two clusters corresponded to alternative reproductive modes (sexual versus asexual)	Gilabert et al. (2009)

SSR, simple sequence repeat; AFLP, amplified fragment length polymorphism; mtDNA, mitochondrial DNA; IBD, isolation by distance.

## Shortcomings of the current applications

Molecular genetics and genomics strategies have proven to be extremely useful tools for understanding pest invasions in many instances, and their utility should not be undervalued. However, a number of limitations to their predictive and explanatory power have become apparent. These shortcomings include limitations in the power of statistical models to accurately reconstruct invasion pathways, particularly because sampling of populations is necessarily incomplete (discussed in detail by Estoup and Guillemaud 2010; Guillemaud et al. 2011). Also, population genetics studies are traditionally based on a limited number of putatively *neutral molecular markers* that are of limited use for studying adaptive processes (reviewed by Kirk and Freeland 2011), which may play a key role in pest invasions (Prentis et al. 2008; Moles et al. 2012). Finally, there can be multiple nonexclusive explanations for observed patterns of genetic structure, and inferences regarding the ecological and evolutionary factors that contribute to pest invasions are subject to interpretation of these patterns (Coop et al. 2010).

While the first two shortcomings listed above have been reviewed in detail elsewhere, the last point deserves some additional attention. The genetic structure of invertebrate pest populations between and within landscapes may be influenced by a myriad of factors (Guillemaud et al. 2011) that can be difficult to tease apart, including (i) species‐specific demographic processes and natural dispersal patterns, (ii) anthropogenic activities (e.g., human‐mediated transport of a pest between different locations; Torriani et al. 2010), (iii) bottom‐up factors (i.e., distribution patterns or genetic structure of hosts; Carletto et al. 2009; Chen and Dorn 2010; Groot et al. 2011), (iv) multitrophic interaction networks including competitive interactions between pests, mutualistic interactions, and interactions between pests and their natural enemies (Lavandero et al. 2011; Wilder et al. 2011), (v) other environmental, landscape, or climatic factors (Chaplin‐Kramer et al. 2011; Sandrock et al. 2011), and/or (vi) adaptive processes (Franck et al. 2007). Contemporary patterns of genetic structure in a pest species will likely reflect the influence of several or all of these factors and their interactions, which may confound the interpretation of the data.

One question that has received increasing attention with regard to invasion biology in recent years is how often and to what extent adaptation promotes pest invasions, yet evidence for adaptation at both the molecular level and the phenotypic level has been notoriously difficult to come by (Barrett and Hoekstra 2011; Verhoeven et al. 2011). An approach that is frequently used to assess the relative role of adaptive evolution in species invasions is to identify sources of invasions and then collect evidence of both genetic and phenotypic differentiation in the invaded compared with the native range of a species (Bossdorf et al. 2005). However, even when parallel genetic and phenotypic differences are observed between native and introduced populations, it is difficult to demonstrate whether such differences have arisen from adaptive processes or from neutral, stochastic events such as founder effects or patterns of multiple introductions and possibly subsequent admixture (reviewed by Keller and Taylor 2008).

Other authors have attempted to identify spatial patterns of genetic structure or allelic variation within an invaded landscape, which are indicative of local adaptation to various selection pressures or climatic gradients. Such patterns can also be difficult to interpret (Coop et al. 2010; and references therein), because the power to identify signatures of selection can depend on the strength of selection, as well as the demographic history of the surveyed populations. For instance, patterns of isolation‐by‐distance or spatial autocorrelation across a landscape can support a number of different hypotheses, including leading‐edge dispersal and range expansion, stepwise introductions (possibly human‐mediated; Torres‐Leguizamón et al. 2011), adaptation to an ecological cline (Telonis‐Scott et al. 2011), or other scenarios, such as multiple introductions of a species from several parts of its native range (Taylor and Keller 2007). For instance, Gilchrist and Meats (2010) examined population genetic structure of the Queensland fruit fly (*Bactrocera tryoni*) across the southern to central parts of its invaded range in Australia. The authors reported a latitudinal cline in microsatellite allele frequencies over a distance of 500 km, but could not determine whether this cline resulted from natural selection or from neutral processes. The latitudinal cline corresponded to differences in monthly average temperatures, decreasing from north to south, and the authors postulated that the cline might also correspond to heritable changes in developmental time or thermal tolerances. However, the two populations at either end of the distribution (north and south) were genetically differentiated source populations that contributed migrants to intermediate populations, and admixture along the north–south gradient generated an allelic frequency cline that could also explain the pattern of isolation by distance.

Similarly, an absence of obvious distance‐related patterns could be caused by factors including geographic barriers to dispersal, patchy distribution of host plants, local adaptation to heterogeneous landscape features, anthropogenic redistribution of the pest, genetic drift (i.e., small population sizes), or factors related to the species' life‐history characteristics. The relative importance of these factors in mediating the distribution and density of invertebrate pests remains difficult to determine. Clearly, molecular genetic data need to be interpreted in the context of additional data, including but not limited to landscape features, climatic variables, and host and natural enemy distribution (Thrall et al. 2011).

## What is new? Recent developments and novel applications

A number of new and ongoing developments are stimulating the application of genetic and genomic tools to study invertebrate pest invasions and outbreaks. First, advances in genomics methodologies promise to provide the double advantage of yielding more detailed and precise information on colonization routes and neutral genetic structure, while also providing the opportunity to identify adaptive loci, and to estimate the contribution of adaptation relative to neutral processes in the spread of invasive pests. We are now able to collect data from thousands of markers from coding and/or noncoding regions of the genome at low cost, using high‐throughput genotyping techniques or through resequencing. Moreover, it is foreseeable that over the coming years, it will become feasible to compare entire genomes of many individuals within and between populations. These technical advances will not solve problems related to adequate and representative sampling nor those associated with the challenges of constructing appropriate models based on realistic scenarios of historical population demography and molecular evolution (Nei et al. 2010). Nonetheless, the availability of genome‐wide sequence data promises an improved understanding of the relative roles of neutral versus adaptive processes (Stapley et al. 2010; Barrett and Hoekstra 2011; Kirk and Freeland 2011) and to tease apart the factors that shape genetic architecture across landscapes. By applying these advances to pertinent questions in carefully designed studies, we will better understand pest invasions. It has been increasingly recognized that both neutral and *adaptive molecular markers* (Kirk and Freeland 2011) should be characterized to develop a more integrated understanding of the processes involved in invasion success.

### Population genomics and adaptation

To develop current and cross‐disciplinary approaches to the study of pest invasions, it is useful to look at case studies from other taxa. A number of methods have been applied to the study of neutral versus adaptive processes in model and nonmodel species over the past several years, and some particularly exciting insights have been generated from work carried out on fish (e.g., Hohenlohe et al. 2010; Whiteley et al. 2011; Jones et al. 2012; Renaut et al. 2012) and plants (e.g., Eckert et al. 2010; Hancock et al. 2011; Strasburg et al. 2012). Understanding the molecular mechanisms underlying range expansion and adaptation often relies on the large amount of data that can be generated through genomics technologies. By incorporating data from thousands of loci, or even entire genomes, we are now much better equipped to capture genetic loci that are both neutral and adaptive.

One strategy that has been applied successfully to several species is the identification of markers, or ‘outlier’ loci, that show different patterns of genetic differentiation between populations compared with the entire set of genotyped loci. Hohenlohe et al. (2010) genotyped single‐nucleotide polymorphisms (SNPs) in threespine sticklebacks (*Gasterosteus aculeatus*) from freshwater and oceanic populations and not only retraced historical routes of colonization between habitats, but also identified genomic regions that had been subject to balancing or divergent selection. Such methods offer great promise both for retracing colonization routes of invasive pests and for identifying genomic regions that are putatively involved in adaptive processes. The complete genomes of 21 *G. aculeatus* individuals derived from numerous pairs of marine and freshwater population were recently sequenced (Jones et al. 2012), and the resulting data provide a number of novel insights into adaptive evolutionary processes. For example, only 17% of regions associated with marine–freshwater divergence contained mutations resulting in changes in amino acid sequences, which suggests that regulatory changes provide an unexpectedly large contribution to adaptive evolution. As is the case with *G. aculeatus*, invertebrate pests are often introduced multiple times to novel habitats and geographic areas and may provide a rich source for other studies of parallel adaption.

Landscape genomics also offers promising applications in the study of invasive invertebrate pests, because adaptive loci can be identified that are associated with environmental and landscape variables (Manel et al. 2010). This approach was used to study the molecular basis of adaptation in loblolly pine (*Pinus taeda*) across its range (Eckert et al. 2010). Allele frequencies at a number of loci were correlated with climatic variables including temperature, accumulated growing degree‐days, precipitation and aridity (Eckert et al. 2010). Based on a comparison with orthologs from the *Arabidopsis* genome, the authors identified a number of candidate genes that may be involved in adaptation to climatic variables including temperature and precipitation. A similar but more comprehensive study of thale cress (*Arabidopsis thaliana*) included 948 accessions derived from the native range of the species, which were genotyped at 215 000 SNPs (Hancock et al. 2011). A number of regions were strongly correlated with one or more of 13 climatic variables, and these provided a set of candidate genes for adaptation to climate. Likewise, landscape genomics may permit the identification of loci from pest species that are associated with adaptation to host plants, climatic variables, or factors associated with anthropogenic landscape management practices, and identifying such loci should lead to a clearer mechanistic understanding of how pests spread over agricultural landscapes.

To derive meaningful information from such large‐scale genomics studies, it is important to carefully select biological systems according to the particular adaptive questions to be addressed. Careful selection of study species and sampling design will minimize problems relating to data interpretation discussed in the ‘Shortcomings of the current applications’ section above. One criterion is the selection of pest species and populations for which natural and demographic histories have been or can be well defined (Nosil and Feder 2012). Additionally, potential selection pressures should be defined *a priori*, and sampling schemes should be established to minimize the confounding effects of environmental effects other than those specifically targeted by the sampling scheme (issues related to sampling are discussed in detail in, e.g., Schoville et al. 2012).

For example, parallel evolution has received considerable recent interest (Radwan and Babik 2012) and is particularly relevant to understanding pest outbreaks because pests are frequently introduced multiple times to different geographic areas over various spatial and temporal scales. Also, they often show evidence of repeated adaptation to similar selection pressures (e.g., commonly used pesticides), and genomics can be used to study the repeatability of evolution. For instance, are only one or a few genes repeatedly involved in adaptation to a particular pesticide, or are different genes involved in adaptation by different populations? To what extent does gene flow between populations mediate the adaptive response and the genomic architecture of divergence between adapted and nonadapted populations (Feder et al. 2012)? To answer these questions, it is important to first identify and sample from multiple nonadapted ‘source’ populations and populations that represent independent examples of the evolution of pesticide resistance. It should be ensured that any other climatic variables, such as temperature and rainfall, vary randomly among source and resistant populations. Finally, potential sources of gene flow between resistant and nonresistant populations should be identified in the field.

### Comparative genomics

The ever‐growing pool of publicly available genomic resources from a variety of species offers opportunities to investigate the molecular basis of adaptation over an evolutionary timescale. There are more than 30 insect species for which complete genome sequences are available, although only a few of these are agricultural pests. Among other species, genomic resources are available for the red flour beetle (*Tribolium castaneum*; full genome sequence; Richards et al. 2008), the pea aphid (*Acyrthosiphon pisum*; full genome sequence; IAGC 2010), the soybean aphid (*Aphis glycines*; partial transcriptome and genome sequence; Bai et al. 2010), and the legume pod borer (*Maruca vitrata*; partial transcriptome sequencing; Margam et al. 2011). Moreover, an ambitious initiative known as i5k was launched in 2011 that aims to sequence and analyze the genomes of 5000 insects and related arthropod species by 2016 (Robinson et al. 2011).

One of the most comprehensive and recent genomics studies of an agricultural pest species was carried out on the two‐spotted spider mite (*T. urticae*), which infests numerous crop species globally, including tomatoes, maize, and soybean (Grbić et al. 2011 and references therein). *Tetranychus urticae* populations are notorious for their ability to adapt rapidly to pesticides. The gene families involved in the digestion, detoxification, and transport of xenobiotics, such as cysteine peptidase genes and glutathione S‐transferase genes, were often expanded in *T. urticae* compared with those known from insects such as *Drosophila* species (Grbić et al. 2011). Moreover, the responsiveness in the expression of a large proportion of these genes to different host plants suggests that they contribute to host adaptation, as well as adaptation to xenobiotics applied for pest control. This finding suggests that repeated duplication of genes involved in detoxification may predispose pest species to rapid adaptation to toxic plant metabolites and commercially produced pesticides.

Another study compared the transcriptomes of two invasive white fly species from the *Bemisia tabaci* species complex (Wang et al. 2011) and identified 24 sequences that showed evidence of divergent positive selection between the two species; these included genes involved in metabolism of carbohydrates, proteins, and xenobiotics, which may play a role in host specialization of the species, as well as the evolution of insecticide resistance.

It is difficult to carry out comparative analyses between the genomes of taxonomically divergent pest species, because mechanisms of adaptation to similar selection pressures (e.g., pesticides) may not involve homologous genome regions among divergent species. Also, large accumulated differences across the genomes of divergent taxa make it difficult to derive meaningful conclusions from such comparisons. Currently, by examining the genomic architecture of adaptation to particular selection pressures (e.g., pesticides) across multiple species, we may be able to draw conclusions about the gene families and mechanisms (e.g., gene duplication) involved in adaptation. However, our ability to use comparative data to understand genomic divergence and convergence will improve with increasing taxonomic coverage of closely related pest species or population pairs. Eventually, placing genomic data in a comparative phylogenetic framework may allow us to identify genomic features that are specific to pest versus nonpest species among different lineages, or to identify genome responses (through e.g., transcript profiling) that are convergent or divergent between ecotypes and species that are subject to similar or different selection pressures (Whitehead 2012). Comparative genomics provides high potential to improve our understanding of the mechanisms behind adaptation to pesticides and other selection pressures.

## Conclusions

It is critical to test alternative hypotheses regarding the factors that promote invertebrate pest invasions (Thrall et al. 2011). A better understanding of evolutionary and demographic processes involved in pest invasions will allow for more rigorous assessment and implementation of management plans (Thrall et al. 2011). Historically, genetic studies of insect pest invasions have focused on retracing routes of introduction, colonization, and spread of pests, and such studies provide valuable information that can be used to minimize the risk of ongoing human‐mediated introductions. However, there are a number of limitations to such studies, in part because the results can be difficult to interpret, and they may not capture critical ecological and evolutionary factors that determine the success of pest invasions.

Recently, increased emphasis has been placed on understanding the biology of invasive invertebrate pests (Pyšek et al. 2010). As a result of advances in statistical modeling and the ongoing development and falling costs of genomics techniques, we are now at a new frontier in terms of our ability to rapidly collect genomic data and to study ecological and evolutionary processes at the molecular level in invasive invertebrate pests. These advances will not solve methodological problems related to limitations in sampling and will not entirely resolve the challenge of interpreting molecular data given complex historical demography. However, they promise to provide more precise information about colonization routes and expand our understanding of adaptive versus neutral processes involved in pest invasions.

Hitherto, these great opportunities have been underutilized in studies of pest invasions and outbreaks. The economic impacts of pests are high, and mitigating these impacts will be a key component of ensuring food security through the coming decades, given the twin challenges of global population growth and climate change.
